# Anti-Fn14 Antibody-Conjugated Nanoparticles Display Membrane TWEAK-Like Agonism ^†^

**DOI:** 10.3390/pharmaceutics13071072

**Published:** 2021-07-13

**Authors:** Ahmed Aido, Olena Zaitseva, Harald Wajant, Matej Buzgo, Aiva Simaite

**Affiliations:** 1 InoCure s.r.o, Department of R&D, Prumyslová 1960, 250 88 Celákovice, Czech Republic; buzgo@labdemo.cz (M.B.); aiva@inocure.cz (A.S.); 2 Division of Molecular Internal Medicine, Department of Internal Medicine II, University Hospital Würzburg, Auverahaus, Grombühlstrasse 12, 97080 Würzburg, Germany; zaitseva_o@ukw.de (O.Z.); harald.wajant@mail.uni-wuerzburg.de (H.W.)

**Keywords:** Fn14, nanoparticles, surface modification, drug-delivery, anti-TNFRSF receptor (TNFR) antibodies

## Abstract

Conventional bivalent IgG antibodies targeting a subgroup of receptors of the TNF superfamily (TNFSF) including fibroblast growth factor-inducible 14 (anti-Fn14) typically display no or only very limited agonistic activity on their own and can only trigger receptor signaling by crosslinking or when bound to Fcγ receptors (FcγR). Both result in proximity of multiple antibody-bound TNFRSF receptor (TNFR) molecules, which enables engagement of TNFR-associated signaling pathways. Here, we have linked anti-Fn14 antibodies to gold nanoparticles to mimic the “activating” effect of plasma membrane-presented FcγR-anchored anti-Fn14 antibodies. We functionalized gold nanoparticles with poly-ethylene glycol (PEG) linkers and then coupled antibodies to the PEG surface of the nanoparticles. We found that Fn14 binding of the anti-Fn14 antibodies PDL192 and 5B6 is preserved upon attachment to the nanoparticles. More importantly, the gold nanoparticle-presented anti-Fn14 antibody molecules displayed strong agonistic activity. Our results suggest that conjugation of monoclonal anti-TNFR antibodies to gold nanoparticles can be exploited to uncover their latent agonism, e.g., for immunotherapeutic applications.

## 1. Introduction

Cancer immunotherapy is a rapidly developing field with a high potential to provide a cure for difficult to treat cancers. Some of the most promising novel immunotherapeutic reagents under consideration target tumor necrosis factor (TNF) receptor superfamily (TNFRSF) receptors (TNFRs) in immune regulation and tumor surveillance [1]. TNFRs and their ligands are, amongst other things, naturally involved in the regulation of innate and adaptive immune responses including natural killer cell activation, T cell co-stimulation, and control of B cell homeostasis [1]. The natural activators of the TNFRs, the ligands of the TNF superfamily, are trimeric transmembrane proteins from which soluble ligand trimers can be released via proteolytic processing [1]. It is noteworthy that many TNFRs, including the immunotherapeutic interesting immune stimulatory receptors CD40, 41BB, CD27, OX40, and TNFR2 and fibroblast growth factor inducible 14 (Fn14), are only robustly activated by their membrane-bound ligands but less or not in soluble form [2]. Thus, there are obstacles in the development of agonistic recombinant soluble TNFSF ligands, including limited agonism, stability, and pharmacokinetics [3,4,5]. Agonistic antibodies targeting TNFRs are therefore considered as useful alternative reagents to activate TNFRs. Unfortunately, IgG antibodies targeting the aforementioned subgroup of TNFRs, which do not or only poorly respond to soluble ligand trimers, typically lack agonistic activity. Instead, FcγR binding, thus a plasma membrane-associated mode of action, is required for these antibodies in order to act as agonists and to stimulate robust receptor signaling [2].

It has been shown that “activating” cell surface anchoring of IgG antibodies using FcγRs can be replaced by fusing antibodies with anchoring domains that recognize cell surface exposed structures distinct from FcγRs [5]. For example, anti-Fn14 antibody fusion proteins with a CD19- or CD20-specific anchoring domain showed strong Fn14 agonism when bound to their anchoring target [5]. Similarly, it has been shown that TNFRs not responding to soluble ligand trimers become properly activated when the molecules are bound to the cell surface, e.g., via chemical means or genetic fusion with an anchoring domain recognizing a membrane-associated target [2,6]. For example, a fusion protein of the soluble Fn14 ligand TWEAK (TNF-like weak inducer of apoptosis) with a FAP-specific single-chain variable fragment (scFv) displays membrane TWEAK-like activity after FAP binding. We hypothesize that the “activating” effect of plasma membrane-associated presentation of anti-TNFR antibodies and TNFSF ligands can be mimicked by grafting these molecules to a solid support. Indeed, it has been found previously that the cell death-inducing TNFR death receptor 5 (DR5) becomes efficiently activated by PGLA and hydroxyethyl chitosan nanoparticles coated with anti-DR5 antibodies [7,8].

Gold nanoparticles (AuNPs) are widely used in different biomedical applications including as a platform for nano-biological conjugates, such as oligonucleotides [9], antibodies [10], and proteins [11]. In addition, the physicochemical and optoelectronic properties of the spherical AuNPs such as surface plasmon resonance, conductivity, large surface-to-volume ratio, excellent biocompatibility, and low toxicity extend the possibilities to exploit them as a new generation of drug delivery systems [12]. All these properties combined make gold nanoparticles a promising tool to deliver the therapeutic agents to the targeted cells. In this work, we utilized gold nanoparticles as a platform to immobilize antibodies against the TNFR Fn14. More specifically, we used a reduction of a gold hydrochlorate solution as a gold nanoparticle synthesis protocol [13] and optimized the (1-ethyl-3-(3-dimethylaminopropyl)carbodiimide hydrochloride)/*N*-hydroxysuccinimide (EDC/NHS) coupling reaction with antibodies, under preservation of their antigen binding abilities. Moreover, in contrast to the free anti-Fn14 antibodies, the AuNPs-immobilized anti-Fn14 antibodies showed strong Fn14 agonism. In this work, we have developed and optimized a method for attaching antibodies to gold nanoparticles and demonstrate the ability of immobilized anti-Fn14 antibodies to trigger proinflammatory receptor signaling.

## 2. Materials and Methods

### 2.1. Materials

Gold (III) chloride acid trihydrate was obtained from VWR International, Stříbrná Skalice, Czech Republic. mPEG-SH/mPEG-Thiol (5 kDa) and SH-PEG-COOH/Thiol-PEG-Acetic Acid (5 kDa) were obtained from Biochempeg, Watertown, MA, USA. Both 38.8 mM trisodium citrate and 1-ethyl-3-(3-dimethylaminopropyl)carbodiimide hydrochloride (EDC) were obtained from Thermo Scientific™, Erlangen, Germany. *N*-hydroxysuccinimide(1-hydroxy-2,5 pyrrolidinedione) (NHS) was obtained from Sigma-Aldrich, Schnelldorf, Germany. 2-(*N*-morpholino)ethanesulfonic acid (MES), anhydrous ≥99% was obtained from VWR chemicals, Darmstadt, Germany. In addition, 50 mM TRIS in 0.33 mg/mL mPEG-SH was used as a blocking buffer. Humira (anti-TNF alpha monoclonal antibody adalimumab) and Cosentyx^®^ (anti-IL-17A monoclonal antibody secukinumab) were a kind gift from Prof. H.-P. Tony (University Hospital Würzburg), and the anti-Fn14 antibodies PDL192 and 5B6 have been described elsewhere [14,15,16]. GpL-TNC-TNF is a fusion protein of soluble TNF with a N-terminal *Gaussia princeps* Luciferase (GpL) domain, which enables detection and quantification of the molecule over several orders of magnitude and has been described elsewhere [17]. The short TNC trimerization contained in the molecule stabilizes the trimeric structure of soluble TNFSF ligands but does not affect their receptor specificity [18,19]. Fn14ed-GpL was generated by cloning the *Gaussia princeps* Luciferase (GpL) without its leader sequence to the C-terminus of the extracellular domain of Fn14.

HEK293, HeLa-RIP3-FADD_KO_, and HT-1080 cells were cultivated in RPMI 1640 medium (Sigma-Aldrich, Schnelldorf, Germany) with 10% fetal calf serum (FCS) (GIBCO, Invitrogen, Merelbeke, Belgium).

### 2.2. Methods

#### 2.2.1. AuNPs Synthesis

The protocol for AuNP synthesis was adapted from [13,20] with slight modifications. In brief, 100 mL of 0.4 mM chloroauric acid solution was boiled in a clean 300-mL glass flask with stir bar. To keep the solution’s volume constant, a reflux column or an aluminum foil was attached on top of the flask. The apparatus was boiled under stirring on a hot plate. One milliliter of a 38.8 mM trisodium citrate solution was added to the chloroauric acid solution resulting in the production of spherical monodisperse gold nanoparticles with a diameter of approximately 60 nm. Au nanoparticles of other sizes were produced by changing the amount of added trisodium citrate and/or the concentration of auric salt within a range between 15 and 100 nm. Upon addition of the trisodium citrate, the color of the solution changed to blue in about 30 s and then to red in another 150 s. The color change during synthesis is attributed to the increase in the size of the gold nanoparticles as the citrate ions reduce the gold ions [21]. The boiling was continued for another 10 min and then cooled to room temperature.

#### 2.2.2. AuNPs Functionalization

To modify the surface of the produced gold nanoparticles with carboxyl groups, a few milliliters of HS-PEG-COOH solution was added to the produced colloidal AuNPs to reach a concentration of 100 μg/mL of HS-PEG-COOH, and then it was mixed for one hour to ensure maximum adsorption on the nanoparticle surface. Carboxyl-modified AuNPs were centrifuged (10,000 RPM for 10 min), and then the pellet was collected in an Eppendorf tube and washed twice with mPEG (0.33 mg/mL) to remove the unreacted trisodium citrate and to ensure a complete cover of the un PEGylated places on the surface of HOOC-PEG-AuNPs and to stabilize the colloidal phase.

#### 2.2.3. AuNPs Antibody Coupling

The HOOC-PEG-AuNPs were then conjugated with the protein of interest according to the EDC-NHS covalent binding procedure adapted from [22,23,24]. Briefly, purified AuNPs were resuspended in activation/coupling buffer (50 mM MES, pH 6.0) and washed with it three times. Then, 24 μL of EDC (200 mM) and 240 μL of NHS (200 mM) were added to 1 mL of the previous solution of AuNPs and incubated with stirring for 30 min at room temperature (RT). After washing particles three times with the activation/coupling buffer to remove the EDC and NHS reagents, 500 μL of the activated AuNPs were incubated with 500 μL of the antibody of interest for two hours at a temperature of 4 °C. The antibody-conjugated AuNPs were washed three times with blocking buffer (Tris 50 mM in mPEG-SH (0.33 mg/mL)) to remove the excess of the unconjugated protein and to block the free activated carboxyl sites on the surface of gold nanoparticles. Finally, the antibody-conjugated AuNPs were resuspended in a blocking buffer for later use. Dot blot analyses revealed that typically approximately 1% *w/w* antibody was conjugated to the AuNPs (Figure S1). For dot blot analyses, a concentration titration series of AuNPs and antibodies were dotted (1 μL) to nitrocellulose. After drying, remaining free binding sites on nitrocellulose were blocked and antibodies were detected via sequential incubation with primary antibody (anti-human IgG primary antibody (H + L) insource: Mouse, Catalog # 31135), (HRP)-conjugated secondary antibodies (Anti-Mouse Immunoglobulins/HRP, #P0260, Source: Rabbit, Dako) and the commercially available ECL western blotting detection reagents and analysis system (Amersham Biosciences, Muenchen, Germany). 

#### 2.2.4. AuNP Characterization

UV-vis: AuNPs samples were collected immediately after synthesis and their optical properties were evaluated via UV–vis spectrophotometry (SpectraMax). The absorption spectra were acquired in the range of 450–650 nm with a step of 5/10 nm.

DLS: The size of the obtained AuNPs (unPEGylated, PEGylated, and grafted with antibodies particles) were analyzed using a Nanophox 123 by dynamic light scattering (DLS) with photon cross-correlation spectroscopy from Sympatec. The particles were purified via centrifugation at 22,000× *g* for 10 min, diluted 100 times in distilled water, and then analyzed. All DLS experiments were carried out at a temperature of 25 °C.

Zetasizer: The effective surface charges on the gold nanoparticles were measured as zeta-potential using a zetasizer from Malvern Instruments Zetasizer, Malvern, UK.

For all measurements, AuNPs were diluted 10–100 times (depending on their concentration) in water.

#### 2.2.5. Binding of *Gaussia princeps* Luciferase (GpL)—Fusion Proteins to Antibodies Immobilized on AuNPs

In order to determine the minimum antibody concentration needed for conjugation, increasing concentrations of easily available anti-TNF or anti-IL17A antibodies were used. The conjugated AuNPs were incubated with a constant amount of GpL-TNC-TNF for 90 min at 37 °C, and after removal of the unbound GpL fusion protein molecules by washing with PBS, AuNPs-associated luciferase activity was measured (see below). For this, particles were transferred to a black 96-well plate and 25 μL of GpL assay solution (1.5 μM Coelenterazin (Carl Roth, Karlsruhe, Germany, 4094.3) in PBS) was added. Luciferase activity was immediately measured (1 s per well) using a PHOMO Photometer (Anthos Mikrosysteme, Schwerin, Germany). Binding values calculated for anti-TNF-AuNPs served as total binding values, while binding values calculated for the anti-IL17A-AuNPs were considered as non-specific binding of GpL-TNC-TNF.

To confirm the coupling of different types of antibodies to gold nanoparticles (AuNPs), the binding of GpL-TNC-TNF or GpL fusion protein of the extracellular domain of Fn14 (Fn14ed-GpL) to the AuNP-immobilized antibodies was determined via equilibrium binding studies. For this, a serial dilution of GpL-TNC-TNF or Fn14ed-GpL was mixed with a constant amount of the antibody-conjugated AuNPs (1 mg/mL in linking solution) for 90 min at 37 °C, and after removal of the unbound GpL fusion protein molecules, AuNP particle-associated luciferase activity was measured. Binding values derived from PDL192-AuNPs or 5B6-AuNPs served as total binding values, while binding values derived from the anti-TNF-AuNPs were considered as non-specific in the case of incubation with Fn14ed-GpL.

Three washing steps were performed to remove unbound proteins. The raw data obtained from the binding studies were analyzed with GraphPad Prism5 software. Total binding values and corresponding non-specific binding values were subtracted to obtain specific binding values. K_D_ values were then calculated with the “nonlinear regression to a one-site specific binding curve” function of GraphPad Prism5.

#### 2.2.6. Determination of IL-8 Production

HT1080 cells (15 × 10^3^/well) were seeded in 96-well tissue culture plates and cultured overnight. The following day, medium was changed and cells were incubated for 16–18 h with the indicated concentrations of AuNP-immobilized anti-Fn14 antibodies, AuNPs, and trimeric soluble Flag-TWEAK with and without anti-Flag oligomerization. In the coculture experiments Fcγ receptor and membrane-bound TWEAK expressing transfectants or HEK293 cells transfected with empty vector as a control were added at a ratio of 1:1 to target cells, and 30 min later, the mixture of cells, containing Fcγ receptor transfectants or HEK293 cells transfected with empty vector, was challenged with anti-Fn14 antibody. At 16–18 h post stimulation, supernatants were collected and analyzed with respect to their IL-8 content using the non-competing anti-IL-8 sandwich antibody pair of the BD OptEIATM human IL8-ELISA kit (BD Biosciences, NJ, USA) according to the manufacturer’s instructions.

#### 2.2.7. p100 to p52 Processing and Western Blotting Analysis

HT1080 cells (1 × 10^6^/well) were seeded in 6-well plates and cultivated overnight. Cells were then treated with AuNPs, anti-Fn14 antibodies, and AuNPs-immobilized anti-Fn14 antibodies. As a positive control, cells were also stimulated with Flag-TWEAK [25]. The next day, cells were collected using a rubber policeman and the remaining medium was removed via two washes with ice-cold phosphate buffered saline (PBS). Total cell lysates were then prepared in 4× Laemmli sample buffer (8% SDS, 0.1 m DTT, 40% glycerol, 0.2 m Tris (pH 6.8), 0.004% bromophenol blue) supplemented with complete protease inhibitor (Roche Applied Science, Grenzach-Wyhle, Germany) and phosphatase inhibitor mixtures I and II (Sigma-Aldrich, Schnelldorf, Germany) via sonication (10 pulses for 20 s) and boiling (95 °C, 5 min). Lysates were cleared via centrifugation (14,000 rpm, 4 °C, 10 min) and separated using sodium dodecylsulfate polyacrylamide gel electrophoresis on 12.5% gels. After transfer to nitrocellulose TRAF1 induction, TRAF2 degradation and p100 to p52 processing were analyzed using western blotting. In brief, free protein binding sites on the nitrocellulose were blocked for 1 h in Tris-buffered saline containing 0.1% Tween 20 and 5% dry milk and proteins of interest were detected via sequential incubation with primary antibodies: (anti-TRAF1 mAb, (45D3) #4715, Source: Rabbit; anti-TRAF2 mAb, Source: Rabbit, (C192), #4724) from Cell Signaling, MA, USA; anti-NFκB p100/p52 mAb, Source: Mouse, #05-361 from Millipore, Darmstadt, Germany; anti-ß-actin mAb, Source: Mouse, clone AC-15, #A1978 from Sigma-Aldrich, Schnelldorf, Germany, horseradish peroxidase(HRP)-conjugated secondary antibodies: (Anti-Mouse Immunoglobulins/HRP, Source: Rabbit, #P0260 from Dako, Hamburg, Germany; Anti-rabbit IgG, HRP-linked Antibody, Source: Goat, #7074, Cell Signaling, MA, USA), and the commercially available ECL western blotting detection reagents and analysis system (Amersham Biosciences, Muenchen, Germany).

#### 2.2.8. Cell Death Assay

Hela-RIP3-FADD_KO_ cells [26] (20 × 10^3^/well) were seeded in 96-well tissue plates. The next day, cells were treated in the presence or absence of TNF (1 ng/mL) with AuNP-immobilized anti-Fn14 antibodies, AuNPs, or Flag-TWEAK. Cell viability was determined after an additional 18 h via crystal violet staining of the remaining attached viable cells to the plate as described elsewhere [27].

## 3. Results

### 3.1. Optimization of Gold Nanoparticles Synthesis Protocol

#### 3.1.1. Controlling the Size and Concentration of Gold Nanoparticles

**Figure 1:**
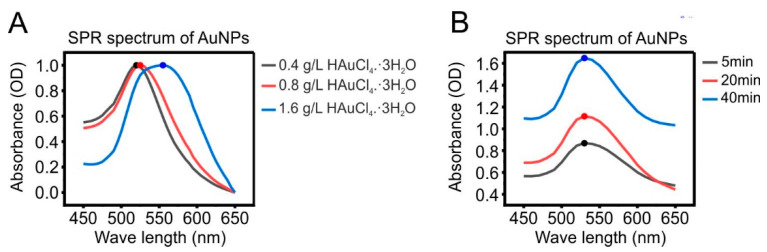
UV-visible absorption spectrum of gold colloids AuNPs prepared with (**A**) different concentrations of HAuCl_4_·3H_2_O, in which increasing the concentration of the gold chloride leads to a lambda max shift from 520 nm to 550 nm, or (**B**) different boiling duration with fixed concentration of HAuCl_4_·3H_2_O, in which the λ_max_ of the different samples was constant at about 530 nm but their λ_max_ absorbance increased according to the increase of the boiling duration.

The size of AuNPs can be adjusted by controlling the concentration of the auric salt (HAuCl_4_·3H_2_O) [20]. To optimize the procedure for the preparation of AuNPs needed for our work, that is in order to obtain AuNPs with the size below 200 nm, we set the trisodium citrate concentration to 38.8 mM and varied the concentration of gold chloride and boiling duration. A concentration of the gold chloride from 0.4 g/L to 1.6 g/L and boiling duration of 10 min was used in the first experiment (Figure 1A). In the second experiment 0.4 g/L of gold chloride was used and the boiling duration was varied from 5 to 40 min (Figure 1B). UV-vis absorption was used to characterize the size of the particles. The wavelength of the maximum absorbance of the plasmon band of the spherical AuNP particles is dependent on the size of the particles [21,28]. As shown in Figure 1A, increasing the concentration of the gold chloride leads to a lambda max shift from 520 nm to 550 nm, indicating an increase of particle size from 15 nm to 80 nm. On the other hand, different results were observed for the influence of the boiling duration. As shown in Figure 1B, the optical density increased with fixed λ_max_ absorbance indicating that the total amount of the produced gold nanoparticles increased with continuing boiling [21]. 

To sum up, our initial experiments demonstrated that the gold nanoparticles size can be easily controlled by changing the initial concentration of the HAuCl_4_. For all further experiments, 0.4 g/L of HAuCl_4_ and a boiling duration of 10 min was used.

#### 3.1.2. Functionalization of the Gold Nanoparticles 

**Figure 2:**
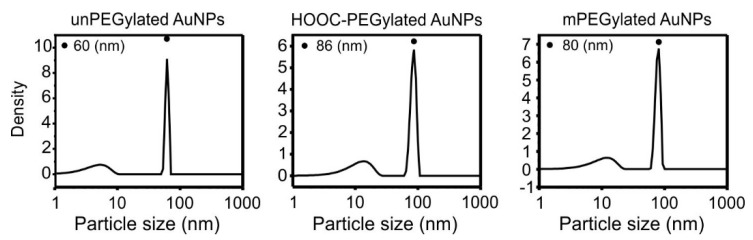
DLS size distributions of colloidal AuNPs unPEGylated (left) and after PEGylation with carboxyl-PEG-SH (center) and mPEG-SH (right)**.** Three reads were performed using the DLS device, and the SD of the results were as follows: AuNPs unPEGylated (0 nm), after PEGylation with carboxyl-PEG-SH (3.5 nm) and mPEG-SH (3.2 nm).

Trisodium citrate plays a role as reducing agent and as stabilizer of the produced gold nanoparticles [29]. The abundance of negative charges of the citrate structure surrounding the surface of AuNPs is known to prevent their aggregation. However, the stabilizing effect of citrate is not significantly enough for storing the particles long term and can be lost after purification. To increase the long-term stability of AuNPs and to introduce chemical groups for the subsequent functionalization, particles were functionalized with a layer of carboxyl- (5 kDa HOOC-PEG-SH) or methoxy- (5 kDa H_3_C-O-PEG-SH) containing polymers. Grafted particles were purified by washing with distilled water to discard the excess of trisodium citrate and free polymer molecules. The particle size after grafting with carboxyl-PEG and methoxy-PEG were then characterized using DLS. As indicated in Figure 2, the increase of particles size after PEGylation with two types of polymers was similar, from ca.60 to ca.86 nm and ca.80 nm, respectively. This is most likely due to the identical molecular weight. The small peak in the range of ±10 nm is a false peak resulting from the effect of the rotational diffusion in the case of the large particles > 40 nm which scatter strongly [30]. To confirm the PEGylation, zeta potential measurements were performed. As shown in Table 1, the PEGylation leads to a change in ζ potential values. In comparison to the citrate stabilization, the charge of the particles after PEGylation with carboxyl-PEG is even more negative (−20 mV), while after the grafting with methoxy-PEG it is less negative (−7 mV). For all further experiments, only particles functionalized with carboxyl-PEG, and later stabilized with m-PEG, were used.

### 3.2. Conjugation of HOOC-PEG-AuNPs with Different Theraputic Antibodies

**Figure 3:**
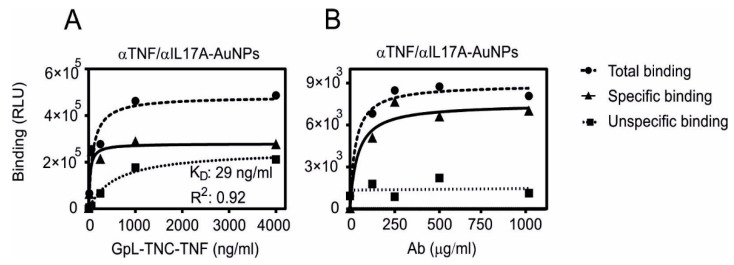
Specific binding of GpL-TNC-TNF to anti-TNF-AuNPs and anti-IL17A-AuNPs. Equal amounts of carboxyl-modified gold nanoparticles (AuNPs; 25 mg/mL) were conjugated with a constant concentration of anti-TNF or anti-IL17A (2 mg/mL). After removal of the excess unbound antibody molecules, the antibody-conjugated AuNPs were incubated with the indicated concentrations of the GpL-TNC-TNF fusion protein for an hour at 37 °C (**A**). Alternatively, increasing concentrations of the two antibodies were used for gold nanoparticle conjugation and were then incubated with a fixed concentration (500 ng/mL) of GpL-TNF-TNC (**B**). After removal of unbound GpL-TNF-TNF molecules, the nonspecific binding values (derived of the anti-IL17A-AuNPs) were subtracted from the corresponding total binding values (derived of the anti-TNF-AuNPs) to obtain specific binding values that were fitted using non-linear regression to a single binding site interaction plot using GraphPad Prism5 software.

Carboxyl-modified gold nanoparticles (HOOC-PEG-AuNPs; ca. 25 mg/mL) were initially conjugated with the commercially available antibodies anti-TNF adalimumab and anti-IL17A secukinumab to establish the coupling conditions. Please note that from now on AuNPs refers to the carboxyl-modified gold nanoparticles. The conjugation process was performed using EDC/NHS coupling. To confirm the post-conjugation functionality of anti-TNF, binding studies using GpL-TNC-TNF were performed. The GpL domain allows the quantification of the binding to the gold nanoparticle-associated antibodies via measurement of the particle-associated luminescence upon removal of the free GpL fusion protein molecules. The binding of GpL-TNC-TNF to anti-TNF-AuNPs was considered as total binding and the binding to anti-IL17A-AuNPs was considered as unspecific binding (Figure 3). A serial dilution of the GpL-TNC-TNF was mixed with a constant amount of the antibody-conjugated AuNPs (1 mg/mL in linking solution). As shown in Figure 3A, specific binding increased with the increasing antigen concentration and reached a plateau with half maximal binding of ~30 ng/mL. These results suggest that the conjugation does not interfere with high affinity binding of TNF to the antibody.

**Figure 4:**
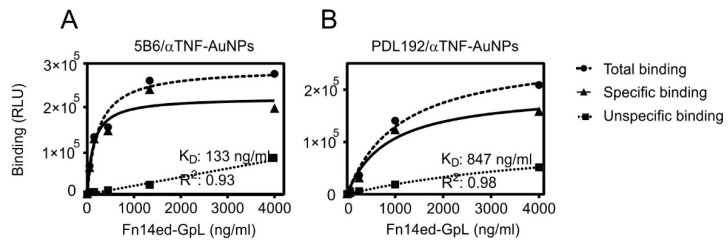
Specific binding of Fn14ed-GpL fusion protein to 5B6-AuNPs and PDL192-AuNPs. Three equal amounts of carboxyl-modified gold nanoparticles AuNPs (25 mg/mL) were conjugated with a constant concentration (2 mg/mL) of the anti-Fn14 antibodies 5B6 (**A**) and PDL192 (**B**) or anti-TNF as a negative control. After removal of unbound antibody molecules, the antibody-AuNP conjugates were incubated with the indicated concentrations of the Fn14ed-GpL fusion protein for an hour at 37 °C. After removal of the unbound Fn14ed-GpL molecules, nonspecific binding values were obtained from the anti-TNF-AuNPs and were subtracted from the corresponding total binding values obtained from the 5B6-AuNPs and PDL192-AuNPs to calculate the specific binding values. The latter were fitted using non-linear regression to a single binding site interaction plot using GraphPad Prism5 software.

In order to evaluate to what extent antibody concentrations used for coupling affect the amount of functional binding sites, increasing concentrations of anti-TNF or anti-IL17A were used for conjugation. The binding with a constant concentration of GpL-TNC-TNF was then determined, as shown in Figure 3B. With a concentration of 250 µg/mL of anti-TNF in the coupling reaction, the maximum amount of functional conjugated antibody was reached and there was no relevant improvement up to 1000 µg/mL. Similar binding studies were then performed with the in-house produced anti-Fn14 antibodies PDL192 and 5B6 and Fn14ed-GpL, a GpL fusion protein of the extracellular domain of Fn14 and GpL (Figure 4).

### 3.3. Fn14 Agonism of Anti-Fn14 Conjugated AuNPs 

To characterize the effect of immobilizing of PDL192 and 5B6 on the gold nanoparticles, in vitro assays were performed to compare the activity of anti-Fn14 AuNPs with those of the soluble antibody variants. Fn14 expression is frequently found to be strongly enhanced in tumor tissue in addition to different tumor cell lines like HT1080 [31,32]. Stimulation of Fn14 results in the activation of the classical NFκB pathway, which leads to production of the inflammatory cytokine IL-8 [15], to enhancement of TNF-induced cell death, and to activation of the alternative NFκB pathway. It was shown that soluble TWEAK stimulates the alternative NFκB pathway and enhances TNFR1-induced cell death but only weakly triggers signaling via the classical NFκB pathway. However, membrane TWEAK induces all these pathways very efficiently [25]. It has been furthermore demonstrated that anti-Fn14 antibodies show no agonistic activity with respect to production of IL-8 and enhancement of TNFR1-induced cell death without oligomerization with protein G or Fcγ receptor binding and also only poorly trigger p100 processing, a hallmark of alternative NFκB pathway activity [15]. We wondered if the AuNP immobilization of the anti-Fn14 antibodies PDL192 and 5B6 can make them agonistic.

To evaluate this, we seeded HT1080 and HT1080-Fn14-knockout cells and challenged the cultures the next day for 14–18 h with dilution series of the following reagents: AuNPs, PDL192/5B6-AuNPs, and Flag-TWEAK with and without anti-Flag. In addition, we analyzed the free antibodies in the presence of FcγR-expressing cells and in comparison to membrane TWEAK-expressing cells. Finally, the supernatants were assayed for IL-8 production using ELISA.

**Figure 5:**
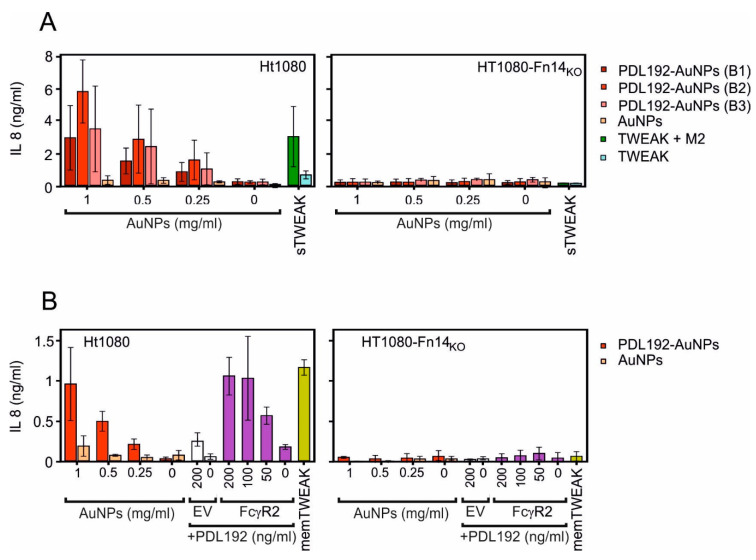
AuNPs-immobilized anti-Fn14 antibody triggers IL-8 production in a similar fashion to cell-anchored antibodies or membrane-bound TWEAK. HT1080 or HT1080-Fn14-knockout cells (15 × 10^3^/well) were seeded in 96-well plates, and the next day, cell culture medium was changed prior to stimulation to reduce the background related to constitutive IL-8 synthesis. Then, cells were stimulated for 14–18 h with a dilution series of AuNPs, AuNPs-immobilized PDL192 (three batches), and Flag-TWEAK with and without crosslinking with the anti-Flag antibody M2. Crosslinking of Flag-TWEAK confers membrane TWEAK-like activity [25] (**A**). Cells were likewise challenged with the anti-F14 antibody PDL192 in the presence of HEK293 cells transfected with a FcγR2-encoding expression plasmid or empty vector (EV) as a control. The HT1080 variants were also challenged with HEK293 cells transfected with a membrane-TWEAK encoding expression plasmid (**B**). The next day, cell supernatants were assayed for IL-8 production using ELISA. Please note, the concentration values of the AuNPs refer to the nanoparticles and not to the amount of conjugated antibody.

Figure 5A shows for three independent batches of AuNP-immobilized anti-Fn14 antibody PDL192 that the immobilized antibody triggered IL-8 production (a hallmark of the classical NFκB pathway) as efficient as anti-Flag oligomerized TWEAK. In the control group with Fn14-deficient HT1080 cells there was no increased IL-8 production confirming the Fn14 specificity of the response analyzed. Likewise, Figure 5B shows that the AuNP-immobilized PDL192 antibody can trigger the same maximal response as high amounts of FcγR-bound antibody and membrane-TWEAK expressing cells.

**Figure 6:**
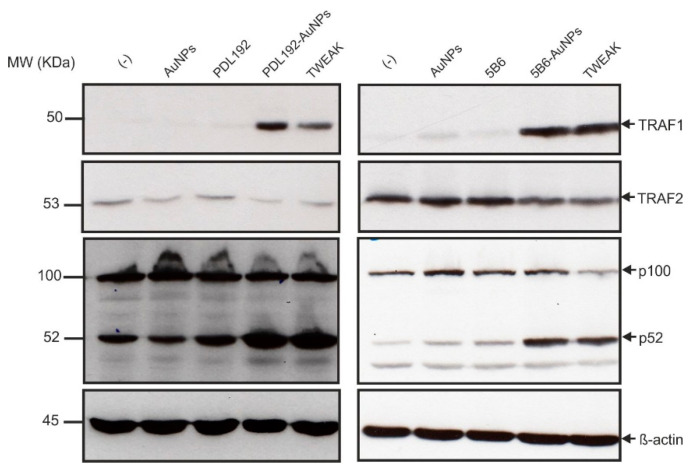
AuNPs-immobilized anti-Fn14 antibodies (PDL192- and 5B6-AuNPs) efficiently trigger p100 processing and induce TRAF1 expression and weak TRAF2 depletion in a similar fashion to sTWEAK. HT1080 cells (1 × 10^6^/well) were seeded in 6-well plates and treated the next day with AuNPs (1 mg/mL), PDL192-AuNPs (1 mg/mL), 5B6-AuNPs (2 mg/mL), PDL192 (400 ng/mL), and 5B6 (400 ng/mL) for 16–18 h. Treatment with Flag-TWEAK (200 ng/mL) served as a positive control. Finally, whole cell lysates were prepared and subjected to western blot analysis to detect TRAF1 induction, TRAF2 degradation, and p100 to p52 processing. Please note, the concentration values of the AuNPs refer to the nanoparticles and not to the amount of conjugated antibody.

To characterize the effect of AuNP-immobilized PDL192 and 5B6 on the alternative NFκB pathway, western blot analysis of whole cell lysate of HT1080 cells stimulated with PDL192- and 5B6-AuNPs, AuNPs, and sTWEAK was performed to detect p100 to p52 processing and, in addition, induction of TRAF1 and degradation of TRAF2, which are associated with alternative NFκB signaling (Figure 6). We observed that AuNP-immobilized anti-Fn14 antibodies but not the soluble antibodies induce TRAF1 expression, p100 processing, and weak TRAF2 depletion in a similar fashion to sTWEAK.

**Figure 7:**
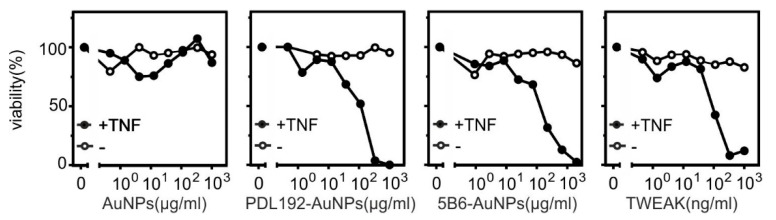
AuNP-immobilized anti-Fn14 antibodies but not control AuNPs enhanced TNF-induced necroptosis in FADD-deficient HeLa-RIPK3 cells. Hela-RIP3-FADD_KO_ cells (20 × 10^3^/well) were seeded in 96-well tissue plates. The next day, cells were treated in the presence or absence of TNF (1 ng/mL) with the indicated concentrations of AuNP-immobilized PDL192, AuNP-immobilized 5B6, AuNPs, or Flag-TWEAK to trigger TNF-induced cell death. Cell viability was determined after 18 h via crystal violet staining. Please note, the concentration values of the AuNPs refer to the nanoparticles and not to the amount of conjugated antibody.

Last but not least, we analyzed the ability of the anti-Fn14-AuNPs to trigger Fn14-mediated enhancement of TNF-induced necroptosis. Similar to p100 processing, the necroptosis-enhancing activity of Fn14 results from its ability to deplete cytoplasmic TRAF2 and so making it less available for the use of other receptors, e.g., TNFR1, which uses TRAF2 to antagonize necroptosis [33,34]. Indeed, AuNP-immobilized anti-Fn14 antibodies but not control AuNPs enhanced TNF-induced necroptosis in FADD-deficient HeLa-RIPK3 cells [26] as well as soluble TWEAK (Figure 7).

In sum, the analyses of Fn14 signaling activities suggest that AuNP-immobilized anti-Fn14 antibodies mimic membrane-bound TWEAK and can therefore be used as Fn14 agonists for therapeutic use.

## 4. Discussion

Fn14 is strongly expressed in most cancers, either by the tumor cells themself or by non-hematopoietic cells of the tumor microenvironment [31]. Fn14 can trigger proinflammatory signaling [31]. Fn14 could therefore considered as an interesting target for immunotherapy of cancer. Recent studies found that free antibodies targeting the TNFRSF receptor Fn14 are largely inactive and only acquire membrane TWEAK-like Fn14-stimulatory activity after oligomerization using protein G or after anchoring of the antibodies to Fcγ receptors, thus after presenting the antibody in a pseudo membrane-bound form [5,15]. Crosslinking by protein G or secondary antibodies as well as FcγR-anchoring, however, is no practicable means for the translational development of Fn14 agonists. We therefore evaluated the possibility to use nanoparticles-immobilized anti-Fn14 antibodies as agonists.

Since the early work of Turkevich and Frens, methods to produce gold nanoparticles in the scale from 9 to 120 nm and with defined size distribution have been optimized for various applications [21]. Gold NPs can be coated with a ligand shell, which provides colloidal stability or allows conjugation with biological molecules via thiols. A method depending on directional covalent binding to a functional polymer on the surface of NPs has been investigated in the literature [35]. One effective method is the use of 1-ethyl-3-(3-dimethylaminopropyl) carbodiimide (EDC) chemistry [36].

We functionalized AuNPs with a carboxyl-containing polymer and then conjugated them with the anti-Fn14 antibodies PDL192 and 5B6 using EDC/NHS chemistry and carried out Fn14 binding studies. The performed binding studies revealed that, even though some antibodies might have coupled through their antigen binding sites, others coupled through antigen-binding irrelevant amino acids and remained functionally active, thus maintaining the ability to bind Fn14 (Figure 4). We show furthermore that the AuNPs-conjugated anti-Fn14 monoclonal IgG1 antibody has an agonistic activity which mimics Fcγ receptor-bound antibodies and membrane-bound TWEAK (Figure 5). This approach will enable promising applications of nanocomposites with antitumor antibodies. Furthermore, we will work on the nano-encapsulation of these nanoparticles to target tumor tissues specifically and prevent the systemic side effects associated with such antibodies.

## 5. Conclusions

Gold nanoparticles of diameter ca. 60 nm have been synthesized via sodium citrate reduction of gold chloride and then functionalized with COOH-PEG-SH to stabilize the colloidal suspension of the gold nanoparticles. We have also demonstrated that the carboxyl-modified gold nanoparticles can be coupled with antibodies of interest using the EDC/NHS coupling procedure. Binding studies with Ab-grafted AuNPs and GpL fusion proteins proved that conjugation of AuNPs with antibodies enables immobilization of antibodies with preservation of a significant antigen binding capacity. More importantly, our findings showed that the conjugation of the anti-Fn14 antibodies PDL192 and 5B6 with gold nanoparticles converted these molecules into potent agonists. Thus, our results suggest that AuNPs can be utilized as a platform to immobilize anti-TNFR antibodies which, on the one hand, help to enhance their agonistic activity in comparison to “free” inactive antibodies by mimicking the effect of cell-anchored antibodies or membrane-bound TNF ligands and, on the other hand, provide the opportunity to develop new generations of drug delivery systems according to their biocompatibility and their tunable synthesis process.

## Figures and Tables

**Table 1:** Comparation of ζ potential values and particles size between the non-PEGylated and PEGylated gold nanoparticles.

Sample Structure	Particles Size	ζ Potential
Trisodium citrate-AuNPs	60 ± 0.2 nm	−14 mV
mPEG-AuNPs	80 ± 2 nm	−7 mV
HOOC-PEG-AuNPs	86 ± 3 nm	−20 mV

## Data Availability

There are no publicly archived datasets.

## Supplementary Materials

The following are available online at https://www.mdpi.com/article/10.3390/pharmaceutics13071072/s1, Figure S1: Dot blot analyses. Concentration titration series of AuNPs, PDL192-AuNPs (three batches) and antibody only were dotted (1 μL) to nitrocellulose. Antibodies were detected by sequential incubation with primary antibody (anti-human IgG primary antibody (H + L)), (HRP)-conjugated secondary antibodies (Anti-Mouse Immuno-globulins/HRP) and the commercially available ECL Western blotting detection reagents and analysis system.
