# Supplementary material for: Anti-Fn14 Antibody-Conjugated Nanoparticles Display Membrane TWEAK-Like Agonism †
Source: Pharmaceutics. 2021 Jul 13;13(7):1072. doi: 10.3390/pharmaceutics13071072 (PMC8308961; doi:10.3390/pharmaceutics13071072)
Supplement: Supplementary file 1 [file pharmaceutics-13-01072-s001.zip › pharmaceutics-1263624-supplementary.pdf]

pharmaceutics

Supplementary Materials: Anti-Fn14 Antibody-Conjugated Nanoparticles Display Membrane TWEAK-like Agonism

Ahmed Aido, Olena Zaitseva, Harald Wajant, Matej Buzgo and Aiva Simaite

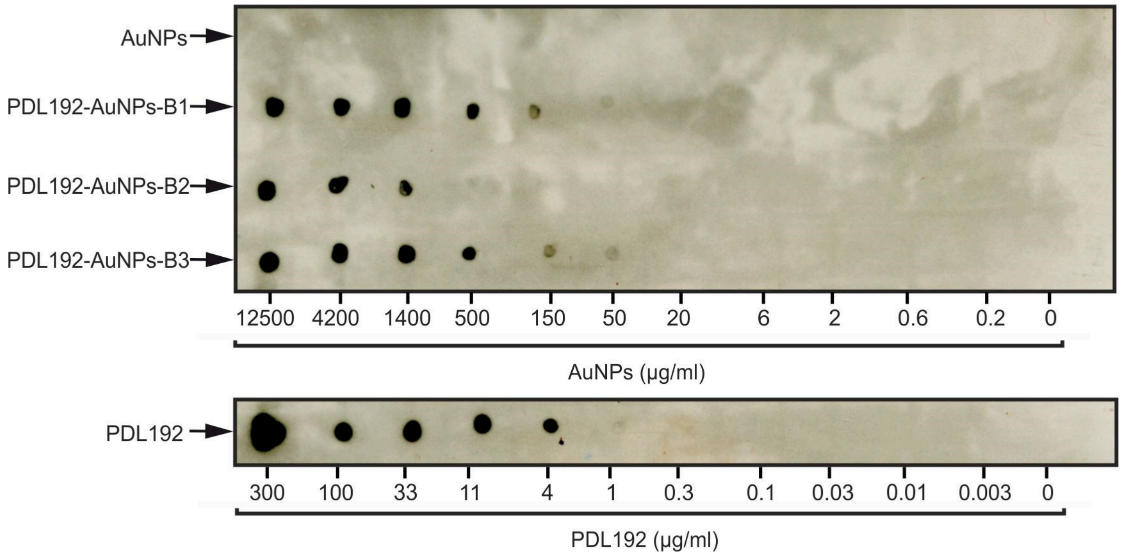

Figure S1. Dot blot analyses. Concentration titration series of AuNPs, PDL192-AuNPs (three batches) and antibody only were dotted (1 µL) to nitrocellulose. Antibodies were detected by sequential incubation with primary antibody (anti-human IgG primary antibody (H+L)), (HRP)-conjugated secondary antibodies (Anti-Mouse Immuno-globulins/HRP) and the commercially available ECL Western blotting detection reagents and analysis system.
